# Herpes zoster risk and burden of disease in immunocompromised populations: a population-based study using health system integrated databases, 2009–2014

**DOI:** 10.1186/s12879-020-05648-6

**Published:** 2020-11-30

**Authors:** Cintia Muñoz-Quiles, Mónica López-Lacort, Javier Díez-Domingo, Alejandro Orrico-Sánchez

**Affiliations:** 1 Vaccines Research Unit, Fundación para el Fomento de la Investigación Sanitaria y Biomédica de la Comunitat Valenciana, FISABIO-Public Health, Avda. Cataluña, 21, 46020 Valencia, Spain; 2 Universidad Católica de Valencia San Vicente Mártir, Carrer de Quevedo, 2, 46001 València, Spain

**Keywords:** Herpes zoster, Immunocompromised, Epidemiology, Population-based study, Healthcare utilization, Vaccine

## Abstract

**Background:**

Estimate the incidence of herpes zoster (HZ), its complications and healthcare utilization rates in adults (≥ 18-years-old) with a wide range of immunocompromised (IC) conditions compared to IC-free cohort.

**Method:**

A population-based retrospective study using the Valencia healthcare Integrated Databases (VID) (2009–2014). HZ and IC were defined using ICD-9 codes in primary care (PC) and hospitalization registers. Incidence rates (IR), risk of HZ, HZ-recurrence, HZ-complications and healthcare utilization rates were estimated in the IC-cohort compared to IC-free.

**Results:**

The study population consisted of 4,382,590 subjects, of which 578,873 were IC (13%). IR (in 1000 persons-year) of HZ overall, in IC and in IC-free cohort was 5.02, 9.15 and 4.65, respectively. IR of HZ increased with age in both cohorts and it was higher for all IC conditions studied, reaching up to twelvefold in subjects with stem cell transplantation. IC subjects had 51% higher risk of developing HZ, 25% higher HZ-recurrence and the risk of HZ-complications was 2.37 times higher than in IC-free. HZ-related healthcare utilization was higher in the IC-cohort than in IC-free (number of hospitalizations 2.93 times greater, hospital stays 12% longer, 66% more HZ-specialist visits, 2% more PC visits, sick leaves 18% longer and 20% higher antiviral dispensation).

**Conclusions:**

Patients suffering from all the IC conditions studied are at higher risk of developing HZ, HZ-recurrence and post-herpetic complications, which implies a substantial morbidity and a high consumption of resources. These results should be considered for vaccine policy implementation.

## Introduction

One in three people aged between 50 and 90 years will develop herpes zoster (HZ) and approximately one in ten will develop postherpetic neuralgia (PHN, defined as dermatomal pain lasting for at least three months after the acute phase of a HZ) [1]. A decrease of the specific cell-mediated immunity against varicella zoster virus (VZV) seems to be the cause of HZ development as a consequence of the VZV reactivation [2]. It could explain the increased risk of suffering HZ in ageing people and especially in people with immunocompromised (IC) conditions due to diseases or treatments that alter the immune response (Immunodeficiency disorders and autoimmune diseases, human immunodeficiency virus (HIV), cancer, organ transplantation) [3–6].

Both, HZ and PHN result in reduced quality of life as well as individual and societal healthcare costs [6–8]. Its treatment, especially that of PHN, is extremely difficult and frustrating for both patients and specialists (who in most cases fail to mitigate the suffering of their patients) [9–11]. Considering these difficulties, a live attenuated vaccine (Zoster Vaccine Live, ZVL) was commercialized in 2006 as a tool to prevent HZ and its complications, and it was licensed in many countries for its use in adults older than 50 years of age [12–14]. Despite its availability, there is relatively scarce use of the current live attenuated vaccine in Spain, where there are efforts to introduce more recommendations for HZ vaccination [15]. The identification and definition of priority HZ at-risk groups is a key step for the design of vaccination programs.

Previous studies on HZ and PHN epidemiology using Real World Data have been developed in order to identify highly susceptible populations such as patients with diabetes, chronic obstructive pulmonary disease (COPD) or heart failure (HF) among others [1, 4, 9, 16–18]. People with IC conditions are among the highest risk of developing HZ [3–5]. However these cohorts cannot benefit from ZVL vaccine due to its contraindication in IC populations [19]. This is the reason why these IC populations have been normally excluded from many epidemiological studies to avoid confusion [1, 16, 17]. As an alternative, an adjuvanted recombinant Zoster vaccine (RZV) has been developed and was first approved in Canada followed by the US in 2017 for its use in adults aged 50 years and older [20, 21]. Clinical studies on immunogenicity, safety and efficacy of RZV have been completed or are ongoing for different IC conditions such as HIV, haematopoietic stem cell transplant (HSCT) or transplant recipients or Inflammatory bowel disease (IBD) [22–25]. Their results will allow broadening the approved indication of RZV [21].

The estimation of the full burden of HZ disease including HZ incidence and recurrence rates, its complications and the evaluation of HZ risk for specific IC subpopulations constitutes a critical step to make an estimation of future impact of the RZV in these risk groups. So far, only a few studies assessed HZ incidence among patients with different IC conditions including populations large enough to allow comparison among the different conditions [3–5, 26, 27]. However, none of them included risk estimations. As recently reviewed by *Mareque* et al., in Spain there is a lack of these types of studies and its heterogeneity and limited populations make it difficult to develop the definition of priority groups for the implementation of a future non-routine HZ vaccination program [15].

The Valencia Region of Spain has a population of around 5 million inhabitants, over 96% of which are insured by the Regional Health System (RHS) [1]. The Region counts with the Valencia health system Integrated Databases (VID), a healthcare electronic databases network gathering real world data (RWD) from hospitalization, primary care, specialist and medication, among other [28]. The quality of VID has been shown in several publications [1, 16, 29–31]. Using the VID, the objective of the present study was to estimate the risk of HZ, its recurrence and complications in adults with a wide range of IC conditions in comparison with the IC-free cohort. Healthcare resources utilization was also assessed.

## Methods

### Study design, population and setting

This is a population-based retrospective cohort study using real-world data from the VID, including a population ≥ 18 years old living in the Valencia Region between 2009 and 2014. The inclusion date was defined as 1st January 2009 for those subjects older than 18 years continuously registered in the RHS for at least 12 months before this date, or first date after 1st January 2009 when the subject was continuously registered in the RHS for at least 12 months before this date and was 18 years old. The date of end of follow-up was defined as end of the study period (31st December 2014) or the date of exit of the RHS (including death), whichever comes sooner.

### Real world data: the Valencia healthcare integrated databases (VID)

We used the following registries from VID [28]: 1) the regional population-based administrative database (SIP), that collects and updates demographic data, health services assignment and usage of the health system; 2) the Ambulatory Care Information System (SIA), that contains medical information for each patient attended in the Primary Care (PC) setting (General Practitioners, GPs, and specialists); 3) the minimum basic data set (MBDS), that collects all diagnosis and procedures from hospitalizations; and 4) the Care Provision Management (GAIA), that gathers drug prescriptions and dispensations data using the Anatomical Therapeutic Chemical Classification System (ATC). Both SIA and MBDS used the International Classification of Diseases, Ninth Revision, Clinical Modification coding system (ICD-9-MC) for codification. All these registries can be linked at the individual level through a unique personal identification number [28, 32].

### Study cohorts

The *IC-cohort* consisted of all eligible individuals with an IC condition-related code registered in SIA or MBDS as follows: HSCT, solid organ transplantation (SOT), haematological neoplasia (HN), solid organ neoplasia (SON), neoplasias overall, HIV, rheumatoid arthritis (RA), systemic lupus erythematosus (SLE), IBD, psoriasis, multiple sclerosis (MS), autoimmune thyroiditis (AT) and immunodeficiency disorders and autoimmune diseases overall (autoimmune) (See supplementary Table 1 for ICD-9-MC codes) [33, 34]. Subjects were considered as IC from the date of the first registry until the end of the follow-up period.

The *IC-free* cohort consisted of subjects with no IC condition-related code detected during the study period. The start of follow-up for the IC-free cohort was the date of inclusion in the study. The IC-free status of an individual changed during the study period if an ICD code for an IC-related condition appeared in MBDS or SIA databases. In the case of PC or hospitalization IC diagnoses, the beginning of IC status was defined as the date in which IC disease was diagnosed and the subject was considered IC from this date until the end of the follow up period (end of the study period or exit from SIP including death).

### Endpoints related to HZ

HZ-incident cases were considered as the first appearance of a HZ-related ICD-code (053.x) [35] in either SIA or MBDS (in any diagnostic position). Any PC medical contact or visit, or hospital admission related to HZ was considered as a medical encounter. In order to ensure that HZ cases were new cases, a period of at least 12 months of registration in RHS was required before the inclusion in the study population.

HZ-recurrent cases were considered when new HZ-related ICD-code appeared at least 6 months after a previous HZ encounter in the same subject. HZ case identification using databases and ICD-codes have shown elevated positive predictive value (92.7%; 95% CI: 89.1–95.4) [32].

HZ-complications were defined as any HZ-related hospitalization with one of the following ICD-codes at discharge in MBDS: central nervous system (CNS) complications (053.0 and 053.1x) which include PHN (053.12, 053.13 and 053.19), ophthalmic complications (053.2x) and other complications (053.7x and 053.8x).

### Statistical analysis

Socio-demographic characteristics (age, gender, calendar year, urban/rural residence, social exclusion risk, health department and comorbidities (diabetes, COPD, HF and chronic kidney diseases (CKD)) of the study population and IC condition type (for the IC cohort), were summarized using descriptive tables including proportions and frequencies.

HZ incidence (number of cases per 1000 persons-year) and recurrence (number of cases per 100 persons-year) rates were estimated for IC population, globally and stratified by gender, age groups: 18–29, 30–39, 40–49, 50–59, 60–69, 70–79 y ≥ 80 years, calendar year and type of IC condition. The respective 95% confidence intervals (CI) were calculated by the exact method of Poisson.

The risk of HZ, HZ recurrence and HZ complications in the IC-cohort subjects respect to IC-free cohort were estimated by a Poisson (quasi-poisson or negative binomial) regression adjusted by age, gender, calendar year, comorbidities (diabetes, COPD, CKD and HF), and health department.

Healthcare resource utilization among IC and IC-free cohorts was estimated as the number of HZ-related PC visits, number and length of HZ-related hospitalizations, number and duration of sick leaves due to HZ and prescriptions and dispensations of antivirals (Aciclovir, Famciclovir and Valaciclovir) during the six months following the HZ diagnosis. We performed different statistical General Linear Models (GLM) to compare the populations with and without IC conditions (see supplementary material 2).

### Ethical considerations

The study protocol was approved by the Ethics Committee of Dirección General de Salud Pública / Centro Superior de Investigación en Salud Pública, which allowed for the linkage of the different databases by the administrators database using a codified SIP number.

## Results

### Study population

The study population included 4,382,590 individuals aged ≥18 years that met the inclusion criteria (49% male). This cohort included 578,873 (13% of the study population) individuals with IC (48% male). Among the IC conditions, neoplasia (7%) was the most frequent, including SON (5.9%) and HN (1.3%). They were followed by psoriasis (3.3%) and RA (0.9%). Diabetes was registered in 11.2% of the population, COPD in 5.1%, CKD in 3.4% and HF in 3.2%. Some conditions were more frequent among men such as HIV (71.4%) or COPD (66.2%) and some other among women including SLE (84.2%), MS (68.9%) or RA (68.2%). Sample size overall and by IC condition in the IC-cohort and IC-free, age distribution and demographic characteristics are listed in Table 1.

**Table 1:** Demographic characteristics for population 18 years old in the Valencia Region from 2009 to 2014 (*n* = 4,382,590)

Characteristics	Population (***n*** = 4,382,590)	IC (***n*** = 578,873)	HIV (***n*** = 13,612)	HSCT (***n*** = 1779)	SOT (***n*** = 18,456)	NEOPLASIAS (***n*** = 306,125)	SON (***n*** = 257,271)	HAEMATOL. NEOP. (***n*** = 55,082)	RA (***n*** = 39,962)
Gender N(%)									
Man	2,145,709 (48.96)	279,122 (48.22)	9716 (71.38)	1030 (57.90)	10,417 (56.44)	159,526 (52.11)	137,305 (53.37)	26,063 (47.32)	12,722 (31.84)
Woman	2,236,881 (51.04)	299,751 (51.78)	3896 (28.62)	749 (42.10)	8039 (43.56)	146,599 (47.89)	119,966 (46.63)	29,019 (52.68)	27,240 (68.16)
Age (years) N									
18–29	1,019,731	62,407	1193	160	1261	11,711	3042	8559	2186
30–39	1,274,301	87,590	4064	276	2741	19,795	9707	9829	4249
40–49	1,243,357	110,019	7630	437	4210	35,539	25,715	10,120	7587
50–59	1,004,914	123,921	4124	628	5018	58,593	49,518	10,063	10,756
60–69	817,757	138,911	1147	602	5347	86,499	76,706	11,562	11,826
70–79	677,388	140,359	476	131	4340	100,993	91,240	12,276	11,391
≥ 80	441,778	98,905	218	14	2209	77,824	70,907	8962	7033
Calendar year N									
2009	4,148,325	264,163	7946	602	9622	129,941	109,293	21,987	22,283
2010	4,088,345	307,912	8555	747	10,680	148,998	123,615	27,113	24,819
2011	4,048,771	349,165	9161	879	11,849	166,335	136,548	31,943	27,275
2012	4,028,926	395,378	9822	1014	13,416	187,925	153,776	36,770	30,207
2013	3,924,744	444,279	10,888	1166	14,907	211,033	171,910	42,451	33,376
2014	3,913,476	494,693	11,810	1312	16,165	234,675	191,360	47,310	36,401
Nationality N (%)	*N **= 4,212,716**	*N **= 563,730**	*N **= 13,136**	*N **= 1703**	*N **= 17,993**	*N **= 293,839**	*N **= 246,025**	*N **= 48,919**	*N = 39,245
Spanish	3,716,485 (88.22)	523,698 (92.90)	11,823 (90)	1618 (95.01)	16,996 (94.46)	275,929 (93.90)	232,726 (94.59)	48,919 (97.36)	35,997 (91.72)
Other	496,231 (11.78)	40,032 (7.10)	1313 10)	85 (4.99)	997 (5.54)	17,910 (6.10)	13,299 (5.41)	1329 (2.64)	3248 (8.28)
Rural N (%)	*N **= 4,380,289**	*N **= 578,747**	*N **= 13,602**	*N **= 1779**	*N **= 18,450**	*N **= 306,032**	*N **= 257,181**	*N **= 55,078**	*N **= 39,958**
Yes	109,358 (2.50)	14,906 (2.58)	216 (1.59)	46 (2.59)	417 (2.26)	8631 (2.82)	7353 (2.86)	1464 (2.66)	1145 (2.87)
No	4,270,931 (97.50)	563,841 (97.42)	13,386 (98.41)	1733 (97.41)	18,033 (97.74)	297,401 (97.18)	249,828 (97.14)	53,614 (97.34)	38,813 (97.13)
Social exclusion N (%)	*N **= 4,021,588**	*N **= 535,996**	*N **= 12,492**	*N **= 1565**	*N **= 17,260**	*N **= 271,137**	*N **= 225,205**	*N **= 51,202**	*N **= 37,974**
Yes	629,179 (15.65)	67,878 (12.66)	3825 (69.38)	157 (10.03)	1894 (10.97)	34,988 (8.72)	16,989 (7.54)	6870 (13.42)	4594 (12.10)
No	3,392,409 (84.35)	468,118 (87.34)	8667 (30.62)	1408 (89.97)	15,359 (89.03)	247,491 (91.28)	208,216 (92.46)	44,332 (86.58)	33,380 (87.90)
**Characteristics**	**SLE (*n*** **= 4878)**	**IBD** **(*n*** **= 28,700)**	**PSORIASIS** **(*n*** **= 139,843)**	**MS** **(*n*** **= 4747)**	**AT** **(*n*** **= 9205)**	**DIABETES (*n*** **= 491,210)**	**COPD** **(*n*** **= 222,902)**	**HF (n = 139,706)**	**CKD (*n*** **= 149,805)**
Gender N(%)									
Man	769 (15.76)	14,599 (50.87)	69,058 (49.38)	1478 (31.14)	1017 (11.05)	258,320 (52.59)	147,456 (66.15)	62,781 (44.92)	82,034 (54.76)
Woman	4109 (84.24)	14,101 (49.13)	70,785 (50.62)	3269 (68.86)	8188 (88.95)	232,890 (47.41)	75,446 (33.85)	76,925 (55.06)	67,771 (45.24)
Age (years) N									
18–29	528	4191	33,887	659	1178	8673	3628	374	2471
30–39	1272	7480	36,959	1661	2337	20,838	7742	903	4722
40–49	1702	8336	35,032	1890	2755	53,081	19,076	2933	7732
50–59	1421	6678	30,709	1344	2751	112,581	39,360	8258	14,114
60–69	952	5512	25,260	822	2011	172,642	65,680	20,223	27,378
70–79	650	4800	17,493	384	1085	193,949	87,338	49,731	52,166
≥ 80	305	2658	8088	113	446	129,895	72,965	83,660	71,404
Calendar year N									
2009	2572	15,898	62,492	3047	3720	347,604	126,850	58,503	53,640
2010	2973	17,772	77,137	3349	4722	365,745	136,093	65,374	61,943
2011	3333	19,513	91,288	3639	6021	378,199	143,759	70,843	71,205
2012	3795	21,777	105,590	3896	6980	393,050	159,700	77,206	80,269
2013	4203	24,326	120,067	4156	7992	404,193	165,847	81,202	88,993
2014	4587	26,890	134,664	4446	9002	413,834	172,506	86,214	98,296
Nationality N (%)	*N **= 4828**	*N **= 28,347**	*N **= 138,860**	*N **= 4678**	*N **= 9172**	*N **= 474,241**	*N **= 213,515**	*N **= 131,310**	*N **= 142,213**
Spanish	4488 (92.96)	26,659 (94.05)	127,492 (91.81)	4353 (93.05)	8673 (94.56)	449,835 (94.85)	203,216 (95.18)	126,428 (96.28)	135,333 (95.16)
Other	340 (7.04)	1688 (5.95)	11,368 (8.19)	325 (6.95)	499 (5.44)	24,406 (5.15)	10,299 (4.82)	4882 (3.72)	6880 (4.84)
Rural N (%)	*N **= 4878**	*N **= 28,694**	*N **= 139,836**	*N **= 4745**	*N **= 9204**	*N **= 491,069**	*N **= 222,830**	*N **= 139,639**	*N **= 149,744**
Yes	91 (1.87)	28,077 (97.85)	3017 (2.16)	112 (2.36)	520 (5.65)	13,830 (2.82)	6758 (3.03)	4938 (3.54)	4136 (2.76)
No	4787 (98.13)	617 (2.15)	136,819 (97.84)	4633 (97.64)	8684 (94.35)	477,239 (97.18)	216,072 (96.97)	134,701 (96.46)	145,608 (97.24)
Social exclusion N (%)	*N **= 4718**	*N = 27,671	*N **= 136,789**	*N **= 4562**	*N **= 9108**	*N **= 447,728**	*N **= 197,306**	*N **= 114,628**	*N **= 126,804**
Yes	3894 (82.53)	23,858 (86.22)	23,130 (16.91)	718 (15.74)	1546 (16.97)	44,835 (10.01)	18,311 (9.28)	4776 (4.17)	7732 (6.10)
No	824 (17.47)	3813 (13.78)	113,659 (83.09)	3844 (84.26)	7562 (83.03)	402,893 (89.99)	178,995 (90.72)	109,852 (95.83)	119,072 (93.90)

*Number of subjects with available information for this category

### HZ incidence rates

Incidence rate (IR) (in 1000 persons-year) of HZ (including PC and hospital registries) was 5.02 in population overall, 9.15 in IC cohort and 4.65 in IC-free cohort. IR of HZ increased with age, in IC-free cohort the lowest IR was 2.26 in 18–29 age group, and the highest IR was 9.54 in ≥80 age group. In IC cohort the lowest IR was 3.61 in 18–29 age group and the highest was 12.93 in 70–79 age group (Table 2).

**Table 2:** Incidence rates of HZ (per 1000 persons - year) by age groups and IC condition in the Valencia Region in 2009–2014

IC condition	Incidence rate of HZ per 1000 PY (95% CI)							
	18–29	30–39	40–49	50–59	60–69	70–79	≥ 80	Overall
**Population**	2.32 (2.27–2.37)	2.46 (2.42–2.51)	2.93 (2.88–2.98)	5.88 (5.80–5.96)	8.63 (8.53–8.74)	9.82 (9.69–9.95)	10 (9.83–10.16)	5.02 (4.99–5.04)
**IC-free cohort**	2.26 (2.21–2.31)	2.36 (2.32–2.41)	2.75 (2.70–2.80)	5.52 (5.44–5.61)	8.12 (8.01–8.23)	9.29 (9.15–9.43)	9.54 (9.36–9.71)	4.64 (4.61–4.67)
**IC-cohort**	3.61 (3.33–3.91)	4.28 (4.02–4.54)	5.52 (5.26–5.79)	9.55 (9.22–9.90)	12.42 (12.05–12.80)	12.93 (12.54–13.33)	12.69 (12.21–13.29)	9.15 (9.02–9.29)
**HSCT**	42.37 (21.89–74.02)	38.94 (24.11–59.53)	50.13 (35.81–68.26)	69.24 (54.08–87.33)	61.82 (47.5–79.1)	51.99 (24.93–95.61)	0	56.07 (48.86–64.04)
**SOT**	6 (3.61–9.37)	6.23 (4.58–8.29)	7.31 (5.83–9.05)	14.89 (12.87–17.14)	17.02 (14.93–19.32)	14.21 (12.06–16.62)	17.26 (13.83–21.29)	12.65 (11.8–13.54)
**HN**	4.96 (4.1–5.94)	4.53 (3.77–5.41)	5.86 (4.97–6.87)	13.95 (12.5–15.52)	18.03 (16.46–19.7)	20.06 (18.37–21.85)	18.44 (16.46–20.6)	11.99 (11.48–12.52)
**SON**	3.87 (2.59–5.56)	4.94 (4.1–5.9)	5.84 (5.25–6.49)	10.24 (9.66–10.85)	12.27 (11.76–12.80)	12.12 (11.64–12.62)	12 (11.43–12.59)	10.97 (10.73–11.22)
**NEOPLASIAS**	4.64 (3.92–5.45)	4.71 (4.14–5.33)	5.86 (5.36–6.40)	10.70 (10.16–11.26)	12.86 (12.40–13.40)	12.91 (12.43–13.39)	12.53 (11.98–13.11)	11.01 (10.79–11.23)
**HIV**	11.9 (8.08–16.89)	12.34 (10.28–14.7)	13.44 (11.96–15.06)	12.8 (10.65–15.27)	13.99 (9.9–19.2)	11.38 (5.68–20.36)	3.87 (0.1–21.56)	12.94 (11.95–13.99)
**AUTOIMMUNE**	3.35 (3.05–3.67)	3.9 (3.62–4.2)	4.7 (4.4–5.01)	8.97 (8.54–9.42)	12.41 (11.87–12.98)	13.98 (13.32–14.66)	13.34 (12.45–14.27)	7.88 (7.71–8.05)
**RA**	4.19 (2.74–6.14)	5.34 (4.1–6.83)	6.03 (5.04–7.16)	10.22 (9.11–11.42)	13.88 (12.65–15.2)	14.3 (13–15.7)	13.71 (12.05–15.53)	11.05 (10.52–11.59)
**SLE**	9.76 (5.33–16.37)	8.62 (5.93–12.1)	8.37 (6.01–11.36)	17.2 (13.27–21.92)	20.37 (15.07–26.94)	21.8 (14.81–30.94)	19.63 (10.73–32.93)	13.36 (11.75–15.14)
**IBD**	4.83 (3.66–6.25)	4.6 (3.78–5.56)	5.16 (4.31–6.12)	9.65 (8.3–11.16)	13.4 (11.59–15.4)	12.97 (11.01–15.18)	16.21 (13.21–19.67)	8.29 (7.77–8.84)
**PSORIASIS**	2.97 (2.63–3.34)	3.37 (3.03–3.74)	3.74 (3.37–4.14)	7.55 (6.98–8.16)	10.39 (9.65–11.16)	12.4 (11.41–13.44)	11.11 (9.72–12.64)	6.13 (5.92–6.34)
**MS**	5.48 (2.51–10.4)	4.39 (2.75–6.64)	4.13 (2.67–6.09)	7.22 (4.87–10.31)	12.34 (8.33–17.62)	10.96 (5.47–19.62)	12.15 (2.51–35.51)	6.33 (5.29–7.51)
**AT**	1.35 (0.37–3.47)	2.07 (1.1–3.55)	4.21 (2.88–5.94)	10 (7.9–12.5)	11.06 (8.42–14.27)	13.56 (9.64–18.54)	17.97 (10.82–28.06)	7.19 (6.31–8.15)

*CI* Confidence interval, *IC* immunocompromised, *HSCT* haematopoietic stem cell transplant, *SOT* solid organ transplantation, *HN* haematological neoplasia, *SON* solid organ neoplasia, *NEOPLASIAS* neoplasias overall, *HIV* human immunodeficiency virus, *AUTOIMMUNE RA* rheumatoid arthritis, *SLE* systemic lupus erythematosus, *IBD* Inflammatory bowel disease, *MS* multiple sclerosis, *AT* autoimmune thyroiditis, *AUTOIMMUNE* immunodeficiency disorders and autoimmune diseases overall (See supplementary Table 1)

**Fig. 1:**
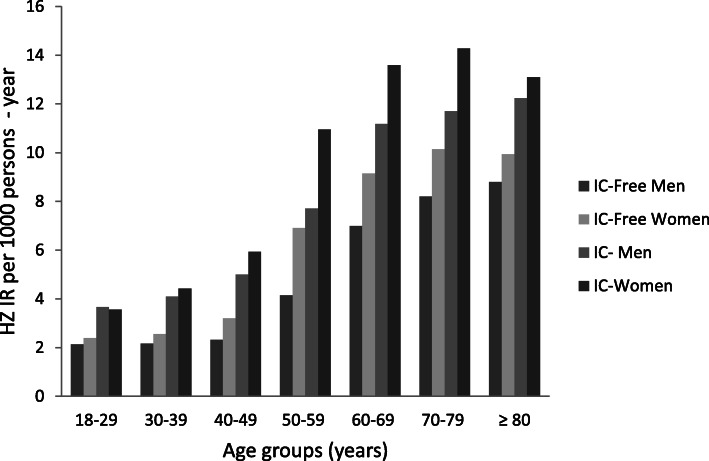
Incidence rates of HZ (per 1000 persons-year) in IC and IC-free cohorts by sex and age groups

IR of HZ remained almost constant throughout the study period with no significant trend in any of the cohorts (not shown). The overall IR of HZ was higher in women than in men in both IC (9.74 vs. 8.48 cases/1000 persons-year) and IC-free cohorts (5.43 vs. 3.83 cases/1000 persons-year) (Fig. 1).

The overall IR (in 1000 persons-year) of HZ in the studied IC conditions ranged from 6.13 for psoriasis to 56.07 for HSCT (Table 2). IR for the other IC conditions were: 13.36 for SLE, 12.94 for HIV, 12.65 for SOT, 11.99 for HN, 11.05 for RA, 10.97 for SON, 8.29 for IBD, 7.88 for immunodeficiency disorders and autoimmune diseases, 7.19 for AT and 6.33 for MS (Table 2).

The adjusted risk of HZ increased by 51% among people with IC conditions with respect to people IC-free (relative risk [RR] 1.51, 95% CrI: 1.48–1.54). It was 33% higher among females than that of males (RR 1.33, 95% CI: 1.31–1.35) and it increased with age (Table 3). The HZ risk was 36% higher for patients with COPD, 12% for diabetes, 16% for HF and 18% for CKD (Table 3).

**Table 3:** Relative risk (RR) estimates and 95% credible intervals (95% CrI) for the association between IC condition and HZ incidence, HZ-complications and HZ recurrence, controlling by sex, age, comorbidities (COPD, diabetes, HF and CKD), health department (as a random effect) and calendar year, using Bayesian Poisson regression

	^**a**^HZ RR (95% CrI)		^**b**^HZ-Complications RR (95% CrI)	^**b**^HZ-Recurrence RR (95% CrI)
**IC**	**IC-free**	1	1	1
	**IC**	1.51 (1.48–1.54)	2.37 (2.01–2.8)	1.25 (1.19–1.31)
**SEX**	**Man**	1	1	1
	**Woman**	1.33 (1.31–1.35)	1.15 (0.98–1.35)	1.19 (1.14–1.24)
**AGE**	**18–29**	1	1	1
	**30–39**	1.06 (1.02–1.09)	5.62 (2.63–11.99)	1.08 (0.95–1.23)
	**40–49**	1.24 (1.2–1.29)	9.71 (4.9–19.24)	1.14 (1.01–1.28)
	**50–59**	2.42 (2.35–2.5)	7.74 (3.95–15.16)	1.21 (1.08–1.36)
	**60–69**	3.43 (3.32–3.54)	12.3 (6.84–22.1)	1.52 (1.36–1.7)
	**70–79**	3.72 (3.61–3.85)	26.14 (14.72–46.44)	1.9 (1.7–2.12)
	**≥ 80**	3.57 (3.45–3.7)	39.72 (22.5–70.12)	2.14 (1.91–2.4)
**COMORBIDITIES**				
**COPD**	**NO-COPD**	1	1	1
	**COPD**	1.36 (1.32–1.4)	2.54 (2.11–3.06)	1.26 (1.17–1.35)
**DIABETES**	**NO-DIABETES**	1	1	1
	**DIABETES**	1.12 (1.09–1.14)	1.07 (0.89–1.28)	1.05 (1–1.11)
**HF**	**NO-HF**	1	1	1
	**HF**	1.16 (1.11–1.21)	2.07 (1.68–2.55)	1.63 (1.39–1.9)
**CKD**	**NO-CKD**	1	1	1
	**CKD**	1.18 (1.13–1.23)	1.63 (1.31–2.03)	1.2 (1.09–1.33)

^**a**^A Quasi poisson regression model adjusted by sex, age, calendar year, health department and comorbidities (COPD, diabetes, HF and CKD) was used to estimate the risk of HZ in IC respect to IC-free subjects

^**b**^A Zero-inflated negative binomial model adjusted by sex, age, calendar year, health department and comorbidities (COPD, diabetes, HF and CKD) was used to estimate the risk of HZ complications (based on hospitalization diagnoses) and recurrence in IC respect to IC-free

### Incidence rates of HZ - related complications

The IR (in 100,000 persons-year) of HZ related complications was 3.63 in population overall, 14.36 in the IC cohort and 2.64 in the IC-free cohort (Table 4). The most common complications were CNS (70.7%) which mainly included PHN (65%), followed by ophthalmic complications (15.93%) and other complications (13.35%).

**Table 4:** Incidence rates of HZ complications (per 100,000 persons - year), overall and by age groups and IC condition

IC condition	Incidence rate of HZ complications per 100,000 PY (95% CI)							
	18–29	30–39	40–49	50–59	60–69	70–79	≥ 80	Overall
**Population**	0.35 (0.18–0.59)	0.75 (0.53–1.04)	1.09 (0.81–1.44)	1.34 (0.99–1.77)	4.34 (3.63–5.15)	10.89 (9.6–12.31)	21.35 (19.08–23.81)	3.63 (3.39–3.88)
**IC-free cohort**	0.33 (0.17–0.58)	0.49 (0.3–0.73)	0.47 (0.29–0.73)	0.78 (0.51–1.15)	2.95 (2.33–3.68)	8.48 (7.25–9.85)	18.78 (16.49–21.3)	2.64 (2.42–2.86)
**IC cohort**	0.58 (0.01–3.21)	5.55 (3.04–9.32)	9.74 (6.57–13.9)	6.91 (4.38–10.37)	14.3 (10.71–18.7)	24.76 (19.83–30.54)	36.12 (28.68–44.89)	14.36 (12.75–16.11)
**HSCT**	310.46 (7.86–1729.79)	161.25 (4.08–898.45)	759.63 (305.41–1565.13)	161.39 (19.55–583)	232.54 (47.95–679.57)	0 (0–1492.1)	0 (0–25,474.82)	300.77 (164.43–504.63)
**SOT**	0 (0–112.66)	38.5 (7.94–112.52)	41.92 (13.61–97.83)	35.98 (11.68–83.97)	90.74 (49.61–152.25)	50.33 (18.47–109.55)	36.08 (4.37–130.34)	50.16 (34.94–69.76)
**HN**	4.13 (0.1–23)	10.68 (2.2–31.22)	40.77 (20.35–72.95)	15.49 (4.22–39.65)	47.43 (25.93–79.58)	51.98 (29.09–85.73)	52.86 (25.35–97.22)	31.8 (24.15–41.11)
**SON**	13.14 (0.33–73.22)	8 (0.97–28.91)	9.69 (3.56–21.1)	6.69 (2.89–13.19)	11.9 (7.46–18.01)	22.86 (16.86–30.31)	36.96 (27.84–48.1)	18.74 (15.79–22.09)
**NEOPLASIAS**	3.1 (0.08–17.3)	7.39 (2.01–18.91)	16.95 (9.49–27.96)	7.66 (3.82–13.71)	14.68 (9.97–20.83)	25.14 (19.14–32.43)	38.84 (29.91–49.6)	19.91 (17.15–23)
**HIV**	0 (0–131.63)	54.1 (19.85–117.76)	37.1 (16.96–70.43)	37.36 (10.18–95.65)	98.64 (20.34–288.25)	95.01 (2.41–529.36)	0 (0–1269.77)	43.2 (27.38–64.82)
**AUTOIMMUNE**	0.71 (0.02–3.96)	3.15 (1.16–6.86)	7.91 (4.52–12.84)	4.25 (1.83–8.37)	15.32 (10.01–22.45)	25.27 (17.39–35.48)	30.81 (19.07–47.09)	10.18 (8.37–12.26)
**RA**	0 (0–58.05)	8.28 (0.21–46.11)	4.5 (0.11–25.08)	3.15 (0.08–17.56)	11.08 (3.02–28.37)	27.05 (12.37–51.36)	40.7 (17.57–80.2)	14.87 (9.53–22.12)
**SLE**	0 (0–244.45)	25.12 (0.64–139.94)	38.74 (4.69–139.92)	24.55 (0.62–136.78)	0 (0–136.77)	61.59 (1.56–343.18)	0 (0–461.25)	25.19 (8.18–58.79)
**IBD**	0 (0–30.57)	4.17 (0.11–23.23)	3.84 (0.1–21.39)	0 (0–18.68)	18.94 (3.91–55.34)	15.59 (1.89–56.3)	29.53 (3.58–106.69)	7.67 (3.51–14.57)
**PSORIASIS**	0 (0–3.86)	0 (0–3.42)	0.99 (0.03–5.52)	2.27 (0.27–8.19)	6.63 (2.15–15.48)	7.88 (2.15–20.17)	35.83 (15.47–70.6)	3.7 (2.26–5.71)
**MS**	0 (0–220.87)	0 (0–71.54)	0 (0–59.58)	0 (0–85.36)	38.62 (0.98–215.15)	0 (0–344.05)	0 (0–1436.88)	4.7 (0.12–26.21)
**AT**	0 (0–123.35)	15.73 (0.4–87.64)	0 (0–47.47)	0 (0–46.07)	0 (0–64.98)	0 (0–117.91)	0 (0–322.51)	2.85 (0.07–15.89)

*CI* Confidence interval, *IC* immunocompromised, *HSCT* haematopoietic stem cell transplant, *SOT* solid organ transplantation, *HN* haematological neoplasia, *SON* solid organ neoplasia, *NEOPLASIAS* neoplasias overall, *HIV* human immunodeficiency virus, *AUTOIMMUNE RA* rheumatoid arthritis, *SLE* systemic lupus erythematosus, *IBD* Inflammatory bowel disease, *MS* multiple sclerosis, *AT* autoimmune thyroiditis, *AUTOIMMUNE* immunodeficiency disorders and autoimmune diseases overall (See supplementary Table 1)

IC patients had double risk of hospitalization by a HZ complication (Table 3). HZ complications risk was 15% higher in women and increased exponentially with age. The risk was approximately double in COPD (vs. NO-COPD) and HF (vs. No-HF) patients and increased by 7% in patients with diabetes and by 63% in CKD population.

### Recurrence of HZ

The recurrence rates (in 100 persons-year) of HZ were 1.66, 2.28 and 1.56 in the population overall, the IC and the IC-free cohorts, respectively. The highest recurrence rate was found in people with HSCT (5.5/100 persons-year), followed by people with HN (3.35/100 persons-year) and HIV (3.2/100 persons-year) (Table 5).

**Table 5:** Recurrence rates of HZ (per 100 persons - year), overall and by age groups and IC condition

IC condition	Recurrence rate of HZ per 100 PY (95% CI)							
	18–29	30–39	40–49	50–59	60–69	70–79	≥ 80	Overall
**Population**	0.99 (0.9–1.09)	1.13 (1.05–1.21)	1.19 (1.11–1.27)	1.31 (1.25–1.38)	1.7 (1.63–1.77)	2.2 (2.12–2.28)	2.71 (2.6–2.83)	1.66 (1.63–1.69)
**IC-free cohort**	0.93 (0.84–1.02)	1.08 (1–1.16)	1.1 (1.02–1.18)	1.23 (1.16–1.3)	1.62 (1.55–1.7)	2.09 (2–2.18)	2.62 (2.49–2.75)	1.56 (1.53–1.59)
**IC cohort**	1.88 (1.44–2.42)	1.63 (1.32–1.99)	1.87 (1.6–2.18)	1.81 (1.61–2.03)	2.08 (1.9–2.27)	2.69 (2.48–2.92)	3.13 (2.84–3.44)	2.28 (2.18–2.38)
**HSCT**	7.38 (1.52–21.56)	2.33 (0.28–8.4)	5.05 (2.18–9.95)	5.39 (2.87–9.22)	5.48 (3.07–9.04)	13.84 (4.49–32.31)		5.5 (4.03–7.34)
**SOT**	2.83 (0.58–8.26)	2.87 (1.15–5.92)	3.12 (1.7–5.23)	2.94 (1.94–4.28)	2.62 (1.8–3.68)	3.55 (2.38–5.1)	3.27 (1.74–5.59)	3.01 (2.5–3.58)
**HN**	3.32 (1.93–5.31)	1.89 (1.01–3.23)	2.04 (1.21–3.23)	3.27 (2.45–4.27)	3.08 (2.42–3.85)	4.16 (3.39–5.04)	4.03 (3.08–5.18)	3.35 (3.01–3.73)
**SON**	1.88 (0.39–5.5)	2.32 (1.27–3.89)	1.82 (1.23–2.58)	1.82 (1.49–2.2)	2.04 (1.78–2.33)	2.46 (2.19–2.75)	3.18 (2.81–3.58)	2.37 (2.22–2.53)
**NEOPLASIAS**	2.93 (1.79–4.52)	1.99 (1.3–2.91)	1.91 (1.41–2.52)	2 (1.69–2.36)	2.17 (1.92–2.43)	2.67 (2.42–2.95)	3.24 (2.89–3.62)	2.5 (2.36–2.64)
**HIV**	3.53 (1.3–7.69)	1.99 (1.11–3.28)	3.01 (2.22–3.99)	3.96 (2.61–5.76)	4.76 (2.38–8.52)	8.39 (2.73–19.59)	0 (0–48.47)	3.2 (2.63–3.85)
**AUTOIMMUNE**	1.65 (1.19–2.24)	1.48 (1.13–1.9)	1.66 (1.33–2.05)	1.62 (1.37–1.91)	2.1 (1.84–2.4)	2.84 (2.5–3.21)	3.1 (2.6–3.66)	2.12 (1.98–2.26)
**RA**	1.57 (0.19–5.67)	1.49 (0.48–3.47)	2.06 (1.18–3.35)	1.68 (1.12–2.43)	2.58 (2–3.28)	2.6 (1.99–3.34)	3.33 (2.42–4.48)	2.43 (2.12–2.77)
**SLE**	0 (0–5.28)	2.73 (0.89–6.37)	3.21 (1.39–6.33)	2.97 (1.42–5.46)	3.23 (1.48–6.13)	1.3 (0.16–4.69)	4.01 (0.83–11.71)	2.75 (1.93–3.79)
**IBD**	1.95 (0.71–4.24)	2.19 (1.16–3.74)	1.58 (0.82–2.77)	1.65 (0.94–2.68)	2.5 (1.64–3.63)	3.49 (2.3–5.07)	4.73 (2.97–7.16)	2.48 (2.06–2.96)
**PSORIASIS**	1.64 (1.06–2.42)	1.36 (0.9–1.96)	1.28 (0.85–1.85)	1.46 (1.1–1.9)	1.71 (1.34–2.16)	2.39 (1.89–2.98)	2.88 (2.04–3.96)	1.77 (1.58–1.97)
**MS**	0 (0–9.86)	1.59 (0.19–5.73)	4.08 (1.5–8.87)	0 (0–2.08)	2.59 (0.71–6.64)	2.89 (0.35–10.44)	0 (0–37.33)	1.94 (1.06–3.26)
**AT**	0 (0–9.6)	2.34 (0.28–8.45)	0.57 (0.01–3.16)	1.75 (0.71–3.61)	1.8 (0.66–3.92)	2.5 (0.81–5.84)	2.97 (0.61–8.69)	1.8 (1.15–2.68)

*CI* Confidence interval, *IC* immunocompromised, *HSCT* haematopoietic stem cell transplant, *SOT* solid organ transplantation, *HN* haematological neoplasia, *SON* solid organ neoplasia, *NEOPLASIAS* neoplasias overall, *HIV* human immunodeficiency virus, *AUTOIMMUNE RA* rheumatoid arthritis, *SLE* systemic lupus erythematosus, *IBD* Inflammatory bowel disease, *MS* multiple sclerosis, *AT* autoimmune thyroiditis, *AUTOIMMUNE* immunodeficiency disorders and autoimmune diseases overall (See supplementary Table 1)

The adjusted risk of developing a recurrent HZ increased by 25% among people with IC conditions respect to people IC-free (RR 1.25, 95% CrI: 1.19–1.31) (Table 3). The HZ recurrence was 26, 5, 63 and 20% higher for people with COPD, diabetes, HF and CKD, respectively.

### Healthcare resources utilization

Overall, HZ-related healthcare resources utilization was higher in the IC-cohort than those IC-free (Table 6): their HZ-related hospitalizations were three times higher (OR 2.93; 95% CI: 2.62–3.27) with 12% longer hospital stays (Mean ratio: 1.12; 95% CI: 1.02–1.23), their HZ-specialized visits were 66% higher (RR 1.66; 95% CI: 1.57–1.76), and they attended 2% more frequently to PC (RR 1.02; 95% CI: 1.01–1.03). In addition, their average of sick leave duration in days was 18% longer (Mean ratio: 1.18; 95% CI: 1.03–1.35) and the antiviral dispensation was 20% higher than in IC-free.

**Table 6:** HZ- healthcare resources utilization by IC subjects in relation to IC-free

	IC
**PC visits for HZ**	**RR (95% CI)**
	1.02 (1.01–1.03)
**Specialist visits for HZ**	**RR (95% CI)**
	1.66 (1.57–1.76)
**Hospitalizations**^a^	**OR (95% CI)**
	2.93 (2.62–3.27)
**Length of hospital stay**	**Mean ratio (95% CI)**
	1.12 (1.02–1.23)
**Sick leave**	**Mean ratio (95% CI)**
	1.18 (1.03–1.35)
**Medication for HZ**	**RR (95% CI)**
	1.2 (1.17–1.22)

^a^Hospitalizations with a HZ CIE-9 code in any diagnostic position; *CI* Confidence interval, *RR* Relative risk, *OR* Odds ratio

## Discussion

This is the first large (> 4 million) population-based study to date investigating the incidence of HZ, its complications and healthcare resources utilization in ≥18-years-old adults in Spain with a wide variety of IC conditions [15]. Our study showed that the adjusted risk of HZ increased by 51% among people with IC conditions. The overall incidence of HZ among individuals with the studied IC conditions considering PC visits and hospitalizations was almost twice as high as the incidence in the IC-free cohort. All the selected IC conditions were above the estimated overall IR in the IC-free cohort, rising up to twelvefold in patients with HSCT. The IR of HZ complications was about 5 times as high in the IC as in the IC-free cohorts. All these findings support that the selected IC conditions increase the risk and severity of HZ episodes and results in higher healthcare resources utilization.

The observed IR of HZ (5.02/1000 persons-year) for the overall study population (4,382,590 individuals aged ≥18 years) is aligned with previous publications [3, 15, 36]. Despite methodological differences such as case definitions or study designs that makes difficult the comparison in absolute values, our findings are consistent with those of other countries studies assessing burden of HZ disease in subjects with different IC conditions, in which there is a strong effect of impaired immune system on the risk of HZ, being HSCT, SLE, HIV or SOT among the most prone to develop a HZ [5, 26, 27]. Concretely in UK, with a study population of 3,698,346, HZ IRs in IC subpopulations raised up to sevenfold for HSCT patients [26], being the most susceptible to HZ as in other publications [3, 5, 27].

In our study, the IC-cohort represented around 13% of the study population. This percentage is difficult to compare among previous studies from different countries in which the values fluctuate from 1.86 in Japan [27] to 16.8 in UK [26] or 25.5–34 in Germany [5], depending on the selected IC conditions, case definitions, observation period and overall methodological heterogeneity. Nevertheless, aligned with these studies, ours reflects that this IC population involves an excesive proportion of the burden of HZ in Spain, with an overall risk of HZ 51% higher than IC-free population. Interestingly, risks estimations showed in our study were modeled using multivariate models. This is a qualitative difference with previous studies [3, 5, 26, 27] where risks estimations were based on rate comparisons, therefore, they are more prone to bias.

Considering all HZ-related complications, we showed that IC patients have more than twice the risk of hospitalization by a HZ complication than IC-free people. 70% of the HZ-related complications were associated to the CNS, with PHN as the most common. In our previous studies we described that 15.7% of the total HZ cases developed in people above 50 years old resulted in PHN lasting for at least three months after the acute phase of a HZ [1, 16, 17]. Note that the impact and burden of PHN in those studies could have been underestimated, as subjects with IC conditions were excluded from the study population. This decision was made to avoid confusion, since the only licensed vaccine at the time was the live attenuated vaccine, which is contraindicated for IC population. Despite the differences regarding population and objectives, these studies are complementary and allow us to conclude that HZ complications pose a great threat to IC patients, worsening their health status and raising the amount of health resources required.

Most of the publications agree that the risk of developing an HZ rises significantly with age, most likely caused by the decreasing cell-mediated immune response to the VZV due to the immunosenescence [32, 37]. This same upward trend is also observed in IC cohort with higher HZ IRs respect to IC-free in all age groups. Interestingly, the IR of HZ in the IC-population aged 50–59 years (9.55 cases/1000 persons-year) was comparable to the one in people aged 80+ in the IC-free group (9.54 cases/1000 persons-year), which indicates that patients with IC conditions are prone to develop HZ at a younger age than individuals IC-free. Age was a variable also associated with an increased risk of suffering HZ hospitalizations, HZ complications and HZ recurrence.

The recurrence rate of HZ ranged from < 1.5 to 6.8% among different studies [36]. Results variations depended on the age, immune status of the study population and, especially, on the follow-up period, being higher in studies with longer follow-up periods [36]. In our study, the Poisson regression analysis showed that the adjusted HZ-recurrence relative risk was 1.25 times higher in patients with IC conditions than in IC-free.

In addition to age and comorbidities, female gender has also shown an increased risk of developing HZ and suffering recurrences and PHN. This result is also in concordance with previous publications [38–40]. It has been hypothesized that it could be due to differences in responses to latent viral infections, which is supported by the gender difference also reported in the incidence of herpes simplex [39]. However, sex differences regarding care-seeking behaviour could be also influencing these results based on secondary data [5]. A clue pointing in that direction could be the almost but not significant differences between men and women when HZ related complications were studied in hospitalization registers in the present study, although more research in this regard is required to draw conclusions.

The greatest strength of our study is, without a doubt, the large study population and the VID, a network of electronic health databases where all the information on each subject (demographic, hospital, PC, specialist, drug prescription and dispensation, etc.) can be linked at the patient level. However, some of its limitations are worth mentioning. Firstly, HZ-related hospitalization rates found are higher than other previously published. It probably represents an overestimation as we considered hospitalizations with HZ diagnoses in any diagnostic position and HZ could represent a secondary diagnosis. Secondly, the proportion of IC population increased during the study period. One of the reasons could be related to IC-cohort case definition, since once a diagnosis of a chronic IC condition was registered the patient remained in the IC-cohort group until the end of the follow-up period. It should also be considered that the individuals with none of the selected IC conditions diagnosed could be not necessarily immunocompetent. On the other hand, severity grade of the studied IC conditions and IC-related therapies have not being considered in this study which may impact the HZ IR and its complications. This lack opens the way for new research in this regard looking for associations between the severity or therapy and time to HZ episode.

As we previously have shown for some other underlying conditions such as Diabetes, COPD, or HF [16, 17], the greater risk of HZ in cohorts with IC conditions is linked to an increase in the utilization of health care resources. They attended more frequently to PC and specialized clinics due to HZ, were hospitalized more often and during longer periods, consumed more antivirals and their sick leaves due to HZ were longer. These data suggest that HZ episodes were more severe in the IC-cohort, contributing to a great reduction in their quality of life and increasing their healthcare-related costs, as previously indicated [41–43].

## Conclusion

In this study we estimate the burden of HZ disease in people with IC conditions in a Spanish Region of 5 million inhabitants. All the IC conditions studied were above the estimated overall IR in the IC-free cohort and the IR of HZ complications was about 5 times as high in the IC as in the IC-free cohorts. In conclusion, the selected IC conditions increase the risk and severity of HZ episodes and result in higher healthcare resources utilization. This first step should be followed by cost effectiveness studies that help us establishing the value of vaccinating these groups of patients when the new prevention strategies become approved in those risk groups.

## Supplementary Information

**Additional file 1: Supplementary Table 1**. CIE-9-MC codes for immunocompromised.

**Additional file 2: Supplementary Table 2**. Generalized lineal models.

## Data Availability

The datasets generated and/or analysed during the current study are available in: https://drive.google.com/drive/folders/11ev5TLPrdJyCDOKgpPuPYdQuZXoj1Wo1?usp=sharing

## Abbreviations

AT: Autoimmune thyroiditis
ATC: Anatomical therapeutic chemical
CI: Confidence interval
CKD: Chronic kidney diseases
CNS: Central nervous system
COPD: Chronic obstructive pulmonary disease
Crl: Credible interval
GAIA: Care provision management (Sistema de información farmacéutica)
GLM: Generalized linear models
HF: Heart failure
HIV: Human immunodeficiency virus
HN: Haematological neoplasia
HSCT: Haematopoietic stem cell transplant
HZ: Herpes zoster
IBD: Inflammatory bowel disease
IC: Immunocompromised
ICD-9-CM: International classification of diseases, Ninth Revision. Clinical modification
IR: Incidence rate
MBDS: Minimum basic data set
MS: Multiple sclerosis
OR: Odds ratio
PC: Primary care
PHN: Post-herpetic neuralgia
RA: Rheumatoid arthritis
ReR: Recurrence rate
RHS: Regional Health System
RR: Relative risk
RWD: Real world data
RZV: Recombinant zoster vaccine
SIA: Ambulatory information system (Sistema de información ambulatoria)
SIP: Personal identification system
SLE: Systemic lupus erythematosus
SON: Solid organ neoplasia
SOT: Solid organ transplantation
VID: Valencia healthcare Integrated Databases
ZVL: Zoster vaccine live
VZV: Varicella zoster virus
