# Supplementary material for: Herpes zoster risk and burden of disease in immunocompromised populations: a population-based study using health system integrated databases, 2009–2014
Source: BMC Infect Dis. 2020 Nov 30;20:905. doi: 10.1186/s12879-020-05648-6 (PMC7708196; doi:10.1186/s12879-020-05648-6)
Supplement: Supplementary file 1 — Additional file 1: Supplementary Table 1. CIE-9-MC codes for immunocompromised. [file 12879_2020_5648_MOESM1_ESM.docx]

Supplementary table 1. CIE-9-MC codes for immunocompromised.

| **Disease or condition** | **CIE-9 codes for disease** | **Description** |
| --- | --- | --- |
| Organ transplantation | V42.x, V58.44, 996.80-996.89, 33.50-33.52, 33.6, 37.51, 41.94, 50.51-50.59, 52.80-52.86, 55.61-55.69  41.0x | Solid organ transplantation or aftercare or complications  Bone marrow or stem cell transplant |
| HIV | V08 | Asymptomatic human immunodeficiency virus (HIV) infection status |
|  | 042 | Human immunodeficiency virus (HIV) disease |
|  | 079.53 | Human immunodeficiency virus type 2 (hiv-2) |
|  | 795.71 | Nonspecific serologic evidence of human immunodeficiency virus (HIV) |
| Neoplasias | 140.0-208.xx  99.25, 99.28 | Malignant Neoplasms  Injection or infusion of cancer chemotherapeutic substance or biological response modifier as an antineoplastic agent |
| Immunodeficiency disorders and autoimmune diseases | 242.00-242.01 | Toxic diffuse goiter (Graves’ disease) |
|  | 245.2 | Chronic lymphocytic tiroiditis (Hashimoto’s disease) |
|  | 279.00-279.9 | Disorders involving the immune mechanism |
|  | 288.00-288.9 | Disease of white blood cells |
|  | 340 | Multiple sclerosis |
|  | 357.0 | Acute infective polineuritis (Guillain Barre Syndrome) |
|  | 358.00-358.01 | Myasthenia gravis |
|  | 555.0-555.9 | Regional enteritis |
|  | 556.0-556.9 | Ulcerative enterocolitis |
|  | 710.0-710.9 | Diffuse diseases of connective tissue |
|  | 714.00-714.9 | Rheumatoid arthritis and other inflammatory polyarthropathies |
|  | 696.x | Psoriasis |
