# Supplementary material for: Herpes zoster risk and burden of disease in immunocompromised populations: a population-based study using health system integrated databases, 2009–2014
Source: BMC Infect Dis. 2020 Nov 30;20:905. doi: 10.1186/s12879-020-05648-6 (PMC7708196; doi:10.1186/s12879-020-05648-6)
Supplement: Supplementary file 2 — Additional file 2: Supplementary Table 2. Generalized lineal models. [file 12879_2020_5648_MOESM2_ESM.docx]

Supplementary table 2. Generalized lineal models

| **Outcome** | **Regression** | **Adjustment** |
| --- | --- | --- |
| Outpatient visits for HZ | Negative Binomial | IS (IC/IS), comorbidity (diabetes, COPD, HF, CKD), Gender, Year, Age and Health Department |
| Specialist visits for HZ | Negative Binomial | IS (IC/IS), comorbidity (diabetes, COPD, HF, CKD), Gender, Year, Age and Health Department |
| HZ hospitalizations  (at least 1  hospitalization = 1/  none = 0) | Binomial | IS (IC/IS), comorbidity (diabetes, COPD, HF, CKD), Gender, Year, Age and Health Department |
| Logarithm hospital stay length (days) for HZ | Lineal | IS (IC/IS), comorbidity (diabetes, COPD, HF, CKD), Gender, Year, Age and Health Department |
| Logarithm of sick leave (days) for HZ | Lineal | IS (IC/IS), comorbidity (diabetes, COPD, HF, CKD), Gender, Year, Age and Health Department |
| Medication for HZ | Negative Binomial | IS (IC/IS), comorbidity (diabetes, COPD, HF, CKD), Gender, Year, Age and Health Department |
